# Supplementary material for: Patient Safety Incidents in Inpatient Psychiatric Settings: An Expert Opinion Survey
Source: Behav Sci (Basel). 2024 Nov 20;14(11):1116. doi: 10.3390/bs14111116 (PMC11591172; doi:10.3390/bs14111116)
Supplement: Supplementary file 1 [file behavsci-14-01116-s001.zip › behavsci-3279921-supplementary.pdf]

Supplementary Material

Table S1. Questionnaire

Section 1 - Demographic	
What is your gender?	• Male<br>• Female<br>• Non-binary<br>• Prefer not to say<br>• Prefer to self-describe
What is your age?	[number from 18 to 100]
In which Country do you primarily carry out your work activity?	[list of countries]
What is your prevalent work setting?	• General hospital<br>• Non-university research structure<br>• Psychiatric hospital<br>• University-research department<br>• University hospital<br>• Other (please specify)
What is your main occupation?	• Clinical job (e.g., clinical psychologist, medical doctor working in direct contact with patients, clinical nurse, etc)<br>• Management job (e.g., clinical risk manager, medical director, human resources manager, etc)<br>• Research job (e.g., professor, researcher, research fellow, etc)<br>• Other (please specify)
What is your profession?	• Medical doctor, psychiatrist/Medical doctor, other<br>• Psychiatric nurse<br>• Nurse, other<br>• Psychotherapist<br>• Psychologist<br>• Mental health counselor<br>• Other (please specify)
How many years of professional experience do you have?	[number from 1 to 100]
How much working time do you spend on patient safety-related activities? (examples of patient safety-related activities: conducting risk assessment, developing safety policies and procedures, monitoring compliance with safety standards, reviewing safety data, training staff on safety protocols, conducting research on patient safety)	• Full time<br>• Most of my working time<br>• At least half of my working time<br>• Less than half of my working time<br>• I do not usually spend working time on patient safety-related activities
According to the WHO, on average in one out of 10 patients an adverse event occurs during hospital care (10% rate). Based on your experience, could you please	[number from 0 to 100]

estimate such a rate (as a percentage) in inpatient psychiatric facilities?	
According to the ERNST Consortium, a second victim is defined as: “Any health care worker, directly or indirectly involved in an unanticipated adverse patient event, unintentional healthcare error, or patient injury, and who becomes victimized in the sense that they are also negatively impacted”. Previous studies have reported that 60% of general nurses and physicians have been a second victim at least one time during their career. Based on your experience, could you please estimate such a rate (as a percentage) for healthcare workers employed in inpatient psychiatric facilities?	[number from 0 to 100]
**Section 2 - Incident Type Categories**	
Based on your experience, please specify the contribution of each incident type category to all the patient safety incidents happening in psychiatric inpatient facilities. The total of the entered values must be 100%.	• Behavior<br>• Blood/blood products<br>• Clinical administration<br>• Clinical process/procedure<br>• Documentation<br>• Healthcare associated infections<br>• Infrastructure/Building/Fixtures<br>• Medical Device/Equipment<br>• Medication/IV Fluids<br>• Nutrition<br>• Oxygen/Gas/Vapour<br>• Patient Accidents<br>• Resources/Organizational Management
**Section 3 - Patient Safety incidents**	
Patient behavior	• Noncompliant/uncooperative/obstructive<br>• Inconsiderate/rude/hostile/inappropriate<br>• Risky/reckless/dangerous<br>• Problem with substance use/abuse<br>• Harassment<br>• Discrimination/prejudice<br>• Wandering/absconding<br>• Intended self harm/suicide<br>• Verbal aggression<br>• Physical assault<br>• Sexual assault<br>• Aggression toward an inanimate object<br>• Death threat
Staff behavior	• Noncompliant/uncooperative/obstructive<br>• Inconsiderate/rude/hostile/inappropriate<br>• Risky/reckless/dangerous<br>• Problem with substance use/abuse<br>• Harassment<br>• Discrimination/prejudice<br>• Wandering/absconding

	• Intended self harm/suicide<br>• Verbal aggression<br>• Physical assault<br>• Sexual assault<br>• Aggression toward an inanimate object<br>• Death threat
Blood/blood products	• Wrong patient<br>• Wrong blood/blood product<br>• Wrong dose or frequency<br>• Wrong quantity<br>• Wrong dispensing label/instruction<br>• Contraindicated<br>• Wrong storage<br>• Omitted medicine or dose<br>• Expired blood/blood product<br>• Adverse effect
Clinical administration	• Not performed when indicated<br>• Incomplete/inadequate<br>• Unavailable<br>• Wrong patient<br>• Wrong process/service
Clinical process/procedure	• Not performed when indicated<br>• Incomplete/inadequate<br>• Unavailable<br>• Wrong patient<br>• Wrong process/treatment/procedure<br>• Wrong body part/side/site
Documentation	• Document missing or unavailable<br>• Delay in accessing document<br>• Document for wrong patient or wrong document<br>• Unclear/ambiguous/illegible/incomplete information in document
Healthcare associated infections	• Bloodstream<br>• Surgical Site<br>• Abscess<br>• Pneumonia<br>• Intravascular<br>• Cannulae<br>• Infected<br>• Prosthesis/Site<br>• Urinary Drain/Tube<br>• Soft Tissue
Infrastructure/Building/Fixtures	• Non existent/inadequate<br>• Damaged/faulty/worn
Medical Device/Equipment	• Poor presentation/packaging<br>• Lack of availability<br>• Inappropriate for task<br>• Unclean/unsterile<br>• Failure/malfunction<br>• Dislodgement/misconnection/removal<br>• User error

Medication/IV Fluids	● Wrong patient<br>● Wrong drug<br>● Wrong dose/strength of frequency<br>● Wrong formulation/presentation<br>● Wrong route<br>● Wrong quantity<br>● Wrong dispensing label/instruction<br>● Contraindication<br>● Wrong storage<br>● Omitted medicine or dose<br>● Expired medicine<br>● Adverse drug reaction
Nutrition	● Wrong patient<br>● Wrong diet<br>● Wrong quantity<br>● Wrong frequency<br>● Wrong consistency<br>● Wrong storage
Oxygen/Gas/Vapour	● Wrong patient<br>● Wrong gas/vapour<br>● Wrong rate/flow/concentration<br>● Wrong delivery mode<br>● Contraindication<br>● Wrong storage<br>● Failure to administer<br>● Contamination
Patient Accidents	● Blunt force<br>● Piercing/Penetrating force<br>● Other mechanical force<br>● Thermal mechanism<br>● Threat to breathing<br>● Exposure to chemical or other substance<br>● Other specified mechanism of injury<br>● Exposure to (effect of) weather, natural disaster, or other force of nature<br>● Falls
Resources/Organizational Management	● Matching of workload management<br>● Bed/service availability/adequacy<br>● Human resource/Staff availability/adequacy<br>● Organization of teams/people<br>● Protocols/policy/procedure/guideline availability/adequacy
Do you think that the provided list of patient safety incidents is exhaustive for inpatient psychiatric facilities?	● Yes<br>● No
Please specify the missing patient safety incidents	[free text]
